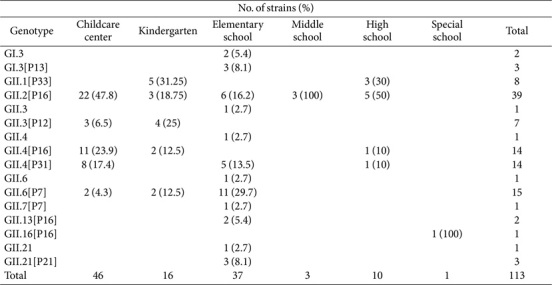# Molecular Epidemiology of Norovirus Outbreaks in Childcare Centers and Schools in South Korea in 2023

**DOI:** 10.4014/jmb.2503.03025

**Published:** 2025-07-18

**Authors:** Won-Jeong Park, Byeong Joon Kim, Doo Won Seo, Yong Chjun Park, Insun Joo, Soo Hwan Suh

**Affiliations:** 1 Food Microbiology Division, National Institute of Food and Drug Safety Evaluation, Ministry of Food and Drug Safety, Cheongju 28159, Republic of Korea; 2 Department of Pharmaceuticals and Biotechnology, Konyang University, Daejeon 36365, Republic of Korea

**Keywords:** Norovirus, outbreak, childcare centers, genotype, VP1, RdRp

## Abstract

Norovirus is a leading cause of acute gastroenteritis and foodborne illness worldwide. In this study, we investigated the epidemiologic and molecular characteristics of norovirus outbreaks in childcare centers and schools in South Korea throughout 2023. A total of 141 stool samples collected from these outbreaks were confirmed positive for norovirus using real-time and conventional RT-PCR, and subsequently analyzed for genotype. The reported outbreaks were most frequently observed in the provinces of Gyeonggi (31.2%) and South Gyeongsang (20.6%), followed by Seoul (12.1%). Outbreaks were most frequently associated with childcare centers (38.3%), primary schools (36.2%), and kindergartens (13.5%). Seasonally, 51.8% of cases occurred during the winter months (November–March), with a peak observed in April. Genotypic analysis revealed that 95.6% of cases were caused by GII norovirus, with the GII.2[P16] genotype being the most prevalent (34.5%). Notably, foodborne transmission was implicated in 13.5% of cases, predominantly involving the GII.2[P16] and GII.6 genotypes. Unlike previous studies that report norovirus genotypes from clinical cases of acute gastroenteritis, our analysis included cases from foodborne outbreaks, thereby offering deeper insights into the role of contaminated food in facilitating norovirus transmission. Furthermore, childcare centers were the primary setting for detection of the GII.4[P16] and GII.4[P31] genotypes, while primary schools exhibited the greatest genotypic diversity, with 12 distinct genotypes identified. These findings indicate a potential shift in norovirus seasonal patterns, with outbreaks extending into late spring. Overall, our results underscore the need for enhanced hygiene practices, robust surveillance systems, and targeted prevention strategies to mitigate norovirus transmission.

## Introduction

Norovirus is a leading cause of viral gastroenteritis worldwide, accounting for over half of all acute gastroenteritis cases each year [1]. Low-income countries bear the brunt of this burden, with acute gastroenteritis linked to more than 25% of deaths in children under 5 in regions such as Africa and Southeast Asia [2]. Norovirus outbreaks frequently occur in confined environments-including hospitals, nursing homes, schools, and childcare centers—with person-to-person transmission being the primary mode of spread [3]. The virus has an incubation period of 24–48 h and is highly contagious, with fewer than 20 viral particles sufficient to cause infection. The main clinical symptoms include nausea, vomiting, abdominal cramps, muscle aches, and diarrhea [1, 4]. Norovirus infections typically peak during the colder months (November to April), although recent studies have shown an increase in hospitalizations for acute gastroenteritis due to norovirus infections among children under 5 during the spring and summer seasons as well [5, 6].

Noroviruses belong to the family *Caliciviridae* and the genus *Norovirus*. Phylogenetic clustering of the capsid gene divides norovirus into six genogroups (GI–GVI) and more than 30 genotypes [7]. In addition to capsid genotyping, analysis of the RNA-dependent RNA polymerase (RdRp) sequence further categorizes norovirus into over 60 P-types [8]. The norovirus genome is approximately 7.5 kb in length, comprising a single-stranded, positive-sense RNA genome with three open reading frames (ORFs): ORF1 encodes nonstructural polyproteins, while ORF2 and ORF3 encode the major capsid protein (VP1) and the minor capsid protein (VP2), respectively [9].

Globally, the GII genogroup is the most prevalent in human infections, with approximately 62% of norovirus foodborne outbreaks attributed to the GII.4 genotype [10]. The GII.4 genotype is dominant worldwide, and the emergence of new GII.4 variants, such as GII.4 New Orleans 2009 and Sydney 2012, is closely associated with global norovirus epidemics [11].

Since the publication of a pivotal study in 2011, increasing epidemiological and molecular evidence has highlighted the emergence of non-GII.4 norovirus genotypes—particularly GII.2, GII.3, and GII.6—as significant etiological agents of acute gastroenteritis among infants and young children [12, 13]. In this study, we conducted a molecular epidemiological analysis of norovirus genotypes associated with acute gastroenteritis and foodborne outbreaks in childcare centers, kindergartens, and schools during 2023, with the aim of elucidating transmission dynamics and providing foundational data to inform targeted prevention strategies.

## Materials and Methods

### Collection of Samples for Norovirus Genotype Analysis

All stool samples used in this study were collected as part of the norovirus surveillance program operated by the Ministry of Food and Drug Safety of the Republic of Korea. The samples were obtained from cases of acute gastroenteritis including norovirus outbreaks that occurred in childcare centers, kindergartens, and primary schools nationwide from January to December 2023. The MFDS classifies outbreaks as group food poisoning when two or more cases are reported in schools, or when fifteen or more cases occur in childcare centers. Accordingly, the associated stool samples were sent to regional public health offices for primary analysis using real-time RT-PCR. Stool samples that tested positive for norovirus were then subjected to secondary analysis using conventional PCR, and if confirmed positive again, Sanger sequencing was performed to verify the presence of human norovirus infection. Confirmed foodborne outbreaks were defined as incidents in which both the etiological agent and the corresponding contaminated food item were identified through epidemiological investigation. Conversely, acute gastroenteritis cases referred to instances where norovirus infection was laboratory-confirmed, but no definitive food source could be established. A total of 141 cases were used for genotype analysis in this study. All primer and probe information, along with the experimental conditions used in this study, are provided in Table 1. The provided stool (rectal swab) samples were mixed with 1 ml of PBS, centrifuged at 100 x g for 1 min, and the supernatant was collected and used as the test solution.

### Sample Preprocessing and Norovirus RNA Detection

Viral RNA was extracted from 280 μl of the stool supernatant using the QIAamp Viral RNA Mini Kit (Qiagen, Germany) according to the manufacturer's instructions. The extracted RNA was then amplified by reverse transcription polymerase chain reaction (RT-PCR) targeting the RdRp-capsid region, which includes portions of ORF1 and ORF2 [14-16]. The primers used for norovirus genetic analysis are listed in Table 1. RT-PCR was performed using the PrimeScript One-Step PCR Kit Ver.2 (TaKaRa Biotechnology Co., Ltd., China). The PCR conditions included a reverse transcription step at 42°C for 30 min, Taq polymerase activation at 95°C for 15 min, followed by 45 cycles of amplification at 95°C, 50°C, and 72°C for 1 min each, a final extension at 72°C for 10 min, and a hold at 4°C [16]. The PCR products were then size-confirmed using a 2% agarose gel in 1× TAE buffer (SeaKem-ME, Lonza Bioscience, USA), producing amplicons of 579 bp for genogroup GI, and 570 bp for genogroup GII.

### DNA Sequencing and Phylogenetic Analysis

For norovirus sequencing analysis, amplified RT-PCR products were purified using the AccuPrep Gel Purification Kit (Bioneer, Republic of Korea). The nucleotide sequences of these purified products were determined using the BigDye Terminator Cycle Sequencing Kit (ABI Prism, Applied Biosystems) using GI- and GII-specific primer sets (GI-MON432/G1SKR and GII-MON431/G2SKR) on an ABI Prism 3500xL Genetic Analyzer (Applied Biosystems, Germany) (Table 1). The resulting sequences were aligned using the MAFFT algorithm, and further analysis was performed with reference to gene information from the National Center for Biotechnology Information (NCBI) GenBank database. Additionally, norovirus genotyping results were confirmed using the automated genotyping tools provided by NoroNet (http://www.rivm.nl/mpf/norovirus/typingtool) and the Human Calicivirus Typing Tool (https://calicivirustypingtool.cdc.gov/). Phylogenetic analysis was conducted using the Maximum Likelihood IQ-TREE method implemented in MegAlign Pro software (Lasergene, v. 18.0, DNASTAR, USA).

## Results

### Epidemiologic Characteristics of Norovirus Outbreaks (Acute Gastroenteritis and Food Poisoning)

**Fig. 1:**
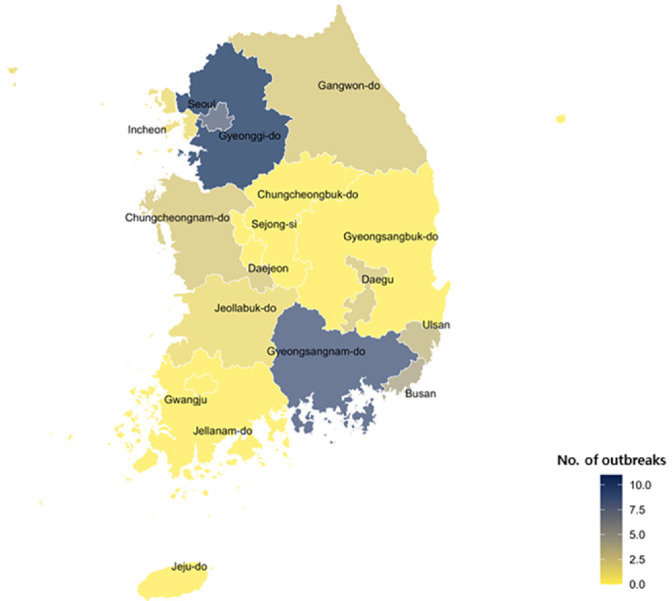
A geographic map illustrating the number of norovirus outbreaks in South Korea in 2023. Darker shades indicate a higher number of outbreaks in the corresponding regions.

From January to December 2023, a total of 141 norovirus-positive cases associated with acute gastroenteritis and food poisoning, as reported to Korea’s Ministry of Food and Drug Safety, were confirmed by real-time PCR. Regionally, the highest number of cases was observed in Gyeonggi Province with 44 cases (31.2%), followed by South Gyeongsang Province with 29 cases (20.6%), Seoul with 17 cases (12.1%), Busan with 13 cases (9.2%), South Chungcheong Province with 12 cases (8.5%), Ulsan and Daegu with 7 cases each (5.0%), North Jeolla Province with 6 cases (4.3%), Gangwon Province with 5 cases (3.5%), and Incheon with 1 case (0.7%) (Fig. 1).

Among these 141 cases, foodborne norovirus infections were identified in 5 outbreaks, accounting for a total of 17 cases. A confirmed foodborne outbreak is defined as an event in which both the causative virus and the implicated food source are identified. The implicated foods were typically multi-ingredient meals prepared and served in group catering settings such as schools or childcare centers. When categorizing the infection sites by facility type, childcare centers accounted for the highest proportion with 54 cases (38.3%), followed by primary schools with 51 cases (36.2%), kindergartens with 19 cases (13.5%), high schools with 12 cases (8.5%), and special schools with 1 case (0.7%).

**Fig. 2:**
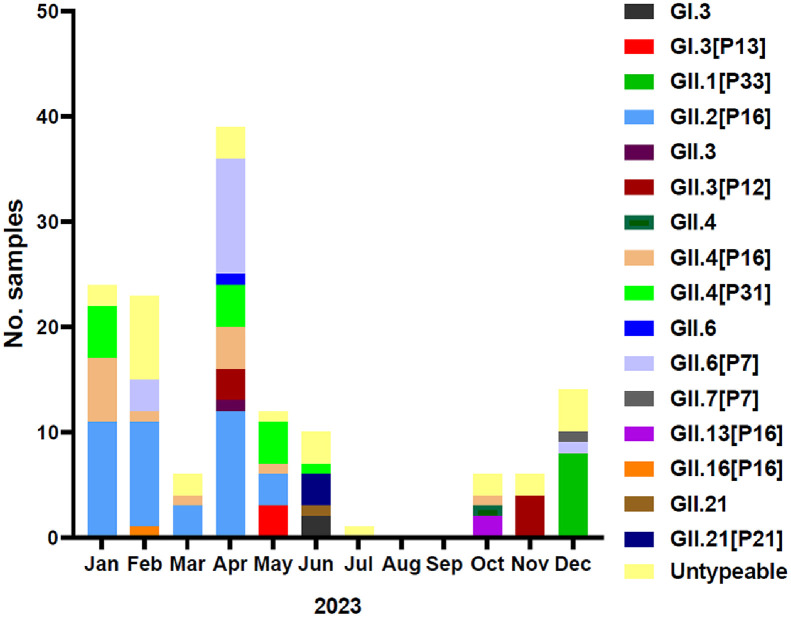
Monthly distribution of norovirus genotypes identified in South Korea in 2023. Genotypes that lack a designated P type and display only the capsid type are classified as P non-typable.

The norovirus cases were predominantly concentrated in the winter season (November to March), which accounted for 51.8% of all cases, with the peak observed in April. Notably, norovirus food poisoning incidents tended to occur primarily between January and April (Fig. 2).

### Phylogenetic Analysis of Norovirus Strains

**Fig. 3:**
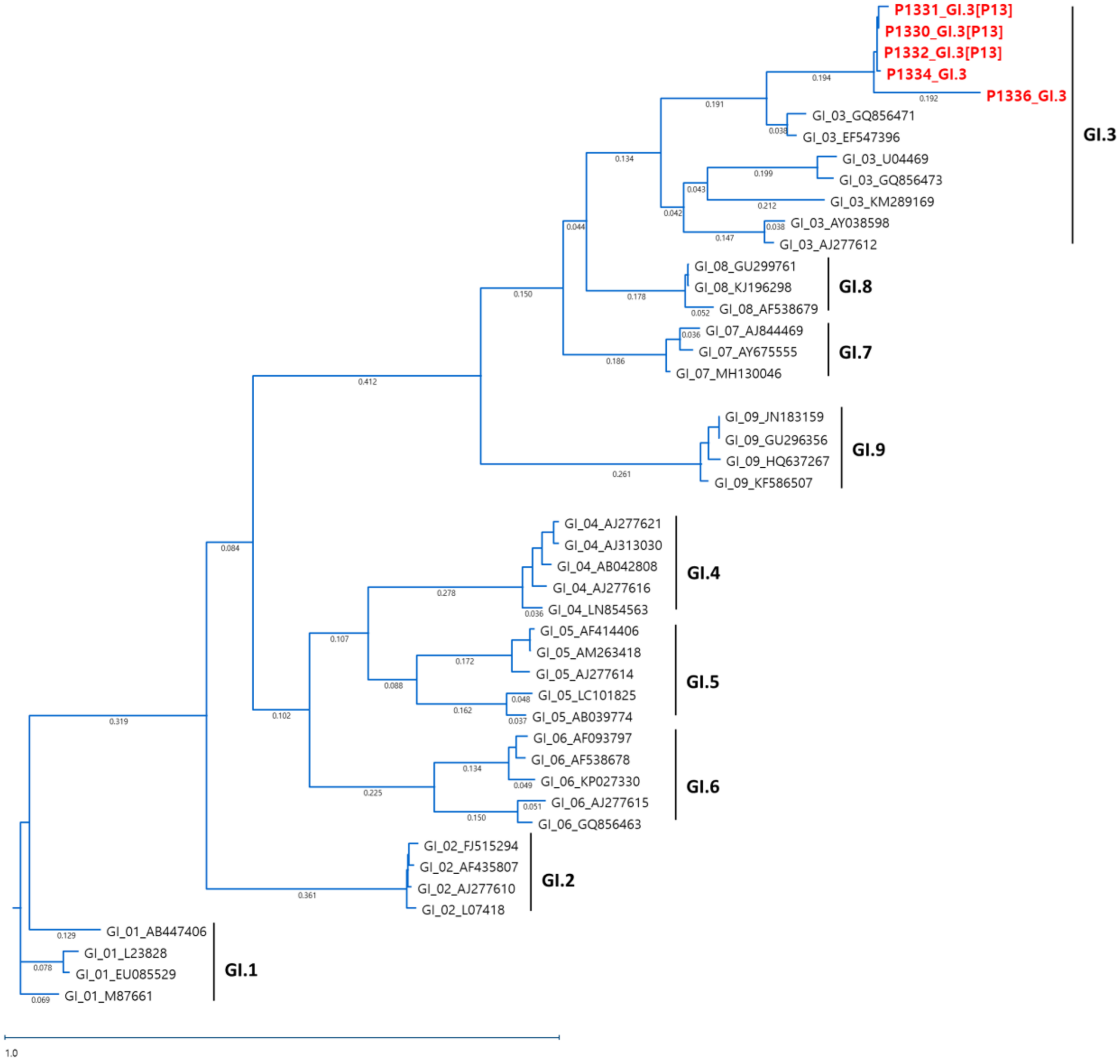
Phylogenetic analysis of Norovirus GI partial capsid and RNA-dependent RNA polymerase (RdRp) nucleotide sequences detected in South Korea in 2023. Strains detected in 2023 are highlighted in red.

Phylogenetic analysis of the ORF1/ORF2 junction sequences of GI norovirus strains demonstrated that the GI.3 strains clustered closely together, indicating limited genetic diversity among the GI genotypes identified during the study period (Fig. 3).

**Fig. 4:**
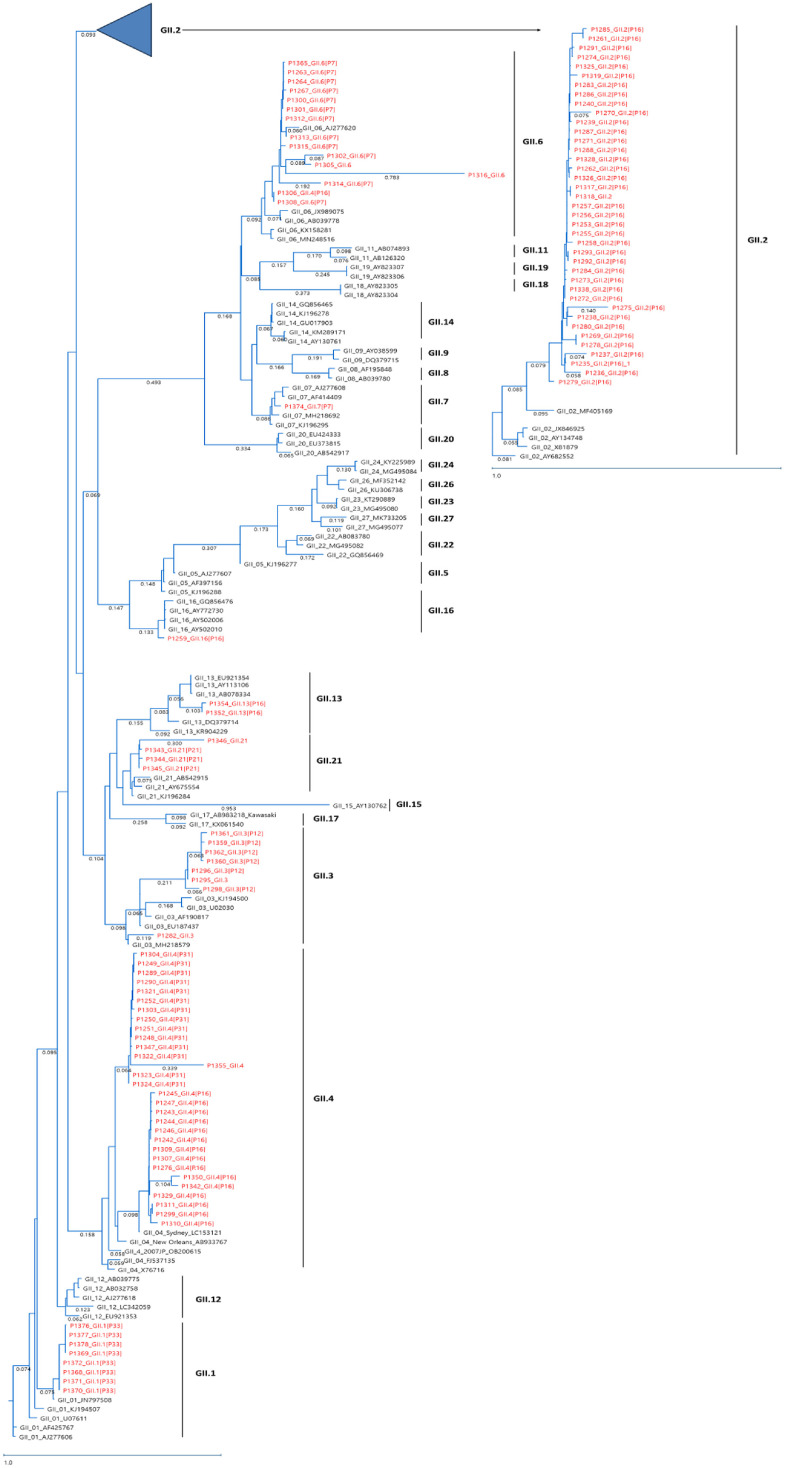
Phylogenetic analysis of Norovirus GII partial capsid and RdRp nucleotide sequences from South Korea in 2023. Strains detected in 2023 are highlighted in red.

Similarly, the phylogenetic tree of GII norovirus strains revealed that the predominant GII.2[P16] strains formed a tight cluster, whereas GII.4[P16] and GII.4[P31] strains exhibited greater genetic variability, suggesting ongoing genetic evolution within the GII.4 lineage (Fig. 4).

### Norovirus Genotype Analysis

Genotype analysis was successfully completed in 113 (92.9%) of the 141 norovirus infection cases. In contrast, in 28 cases (17.8%), although the samples tested positive by real-time RT-PCR, conventional RT-PCR yielded negative results, so the genotype could not be determined. Of the 113 cases with confirmed genotypes, 107 were successfully characterized in both the capsid region (ORF2) and the RNA-dependent RNA polymerase region of ORF1, whereas the remaining 6 were genotyped only for the capsid region.

Genotype analysis revealed that 5 cases (4.4%) belonged to the GI genotype and 108 cases (95.6%) to the GII genotype, confirming that GII norovirus was the predominant genotype. Among the GI genotypes, GI.3[P13] was detected in 3 cases (2.7%) and GI.3 (P-untypable) in 2 cases (1.8%). Within the GII genotypes, GII.2[P16] was the most common, accounting for 39 cases (34.5%), followed by GII.6[P7] in 15 cases (13.3%), GII.4[P16] in 14 cases (12.4%), and GII.4[P31] in 14 cases (12.4%). Additionally, GII.1[P33] was detected in 8 cases (7.1%), GII.3[P12] in 7 cases (6.2%), GII.21[P21] in 3 cases (2.7%), and GII.13[P16] in 2 cases (1.8%). Genotypes detected in only one case each included GII.3, GII.4, GII.6, GII.7[P7], and GII.16[P16]. Notably, among the norovirus food poisoning cases, GII.2[P16] was the predominant genotype, being detected in a total of 16 cases (Table 2).

### Norovirus Acute Gastroenteritis Molecular Epidemiology: Genotype Distribution

Norovirus genotypes were analyzed by categorizing facilities that provide group meals by age, namely, daycare centers, kindergartens, elementary schools, middle schools, and high schools. According to the age group-based analysis of norovirus prevalence, the highest proportion was observed in childcare centers (40.7%), followed by elementary schools (32.7%), kindergartens (14.2%), high schools (8.8%), middle schools (2.7%), and others (0.9%). GII.2[P16] emerged as the predominant genotype across all settings. In particular, among norovirus cases in daycare centers, GII.2[P16] accounted for the highest proportion at 47.8%, followed by GII.4[P16] at 23.9%, and GII.4[P31] at 17.4%. In daycare centers, the GII.4 genotypes (GII.4[P16] and GII.4[P31]) were detected in 19 out of 29 cases (65.5%), indicating that daycare centers are a major site for GII.4 genotype detection. Meanwhile, the greatest genotype diversity was observed in elementary schools, where a total of 12 distinct genotypes were detected, thereby highlighting significant genetic variation (Table 3).

## Discussion

In communal settings such as daycare centers, kindergartens, and schools, norovirus infections can rapidly spread through communal meals, underscoring the importance of proper hygiene management and infection surveillance. We sought in this study to identify the major norovirus genotypes in domestic outbreaks by analyzing specimens collected from food poisoning and gastroenteritis patients—particularly among groups vulnerable to norovirus—and to investigate the virus’s molecular epidemiology.

In areas where interpersonal contact is frequent or population density is high, person-to-person transmission serves as a major risk factor, and the frequency of norovirus infections tends to increase with higher contact rates [17]. The Seoul metropolitan area, comprising Seoul, Incheon, and Gyeonggi Province, is home to over 50% of the nation’s population (https://kosis.kr/visual/populationKorea/PopulationDashBoardMain.do (KOISIS)), the density of which is considered to be the primary reason for the elevated incidence of norovirus infections in the region. In fact, the metropolitan area accounted for 44% of all cases, with particularly high rates observed in Seoul and Gyeonggi Province.

Norovirus is known to have a higher infection rate among young children under 5 years of age. In these immunologically immature individuals, infection can lead to severe dehydration and exacerbated gastroenteritis symptoms, potentially progressing to critical illness or even death [18, 19]. Our analysis of age-specific infection rates revealed that children aged 0–7 years attending daycare centers and kindergartens accounted for 54.8% of all cases, highlighting that young children—who often live in densely populated environments and possess relatively lower immunity—are particularly susceptible to norovirus infections.

Norovirus typically exhibits seasonal characteristics, with outbreaks most common during the winter months (December to February). In China, for instance, the highest incidence of norovirus has been reported between October and March [15, 20, 21]. However, in 2021, the highest detection rate in domestic daycare centers was observed in April, and cases were even reported during the summer months (July–August). This deviation from the traditional seasonal pattern suggests that, in Korea, the peak detection period has shifted from December–January to later in the spring (May), thereby extending the outbreak season [22]. Similarly, a seasonal analysis of acute gastroenteritis cases in European children found that infections occurring between June and July accounted for 50% of cases [5].

Consistent with this trend, our study found that 51.8% of cases occurred during the winter season (November to March), with the highest detection rate recorded in April. This pattern mirrors the recent shifts observed in the timing of norovirus outbreaks in Korea. Notably, the concentration of foodborne norovirus outbreaks between January and April reflects both our findings and recent changes in detection patterns. In Korea, most childcare centers, kindergartens, and schools observe extended winter breaks in January and February, with the new academic term typically beginning in March. These findings imply that the increase in norovirus cases observed in March and April may be associated with the resumption of academic activities following the winter break, a period characterized by heightened interpersonal contact within educational environments compared to the relatively inactive months of January and February. These alterations in seasonal distribution underscore the need for continuous surveillance and research to elucidate the underlying causes and to develop effective prevention and response strategies.

A total of 19 foodborne norovirus cases were detected in our dataset, accounting for 13.5% of all cases, which closely aligns with the global average of 13.7% for foodborne norovirus infections reported by CalciNet and Noronet [23]. GII.2[P16] was the most frequently identified genotype in foodborne outbreaks in our study. While the GII.4 genotype commonly spreads via person-to-person transmission [24], GII.2[P16] is notable for its high transmissibility and potential to cause significant foodborne outbreaks [25, 26]. This finding offers valuable insights for tailoring prevention and management strategies to different transmission routes.

Recent studies from Thailand and Japan similarly report GII.2 and GII.2[P16] as predominant genotypes, especially among children in kindergartens and schools [27-29]. The global emergence of GII.2[P16] norovirus variants has been linked to shifts in the epidemiological patterns of norovirus infections. Between 2016 and 2018, these strains contributed to increased outbreaks in regions such as China, Germany, and Japan, temporarily replacing the dominant GII.4 strains [26, 32]. In Taiwan, a sharp rise in GII.P16-GII.2 outbreaks was noted in late 2016, primarily in kindergartens and elementary schools [33]. Our findings are consistent with previous reports, with GII.2[P16] identified as the predominant genotype in norovirus outbreaks at Korean educational institutions. This supports its strong association with infections in younger populations. Phylogenetic analysis showed that GII.P16-GII.2 strains have a relatively high basic reproduction number (R_0_ ≈ 1.99) and are under positive selection in the VP1 region [26], suggesting increased transmissibility and immune evasion potential. Given the rapid global spread and frequent involvement of emerging GII.2 lineages in outbreaks, continued molecular surveillance is warranted to monitor their evolution and public health impact.

One of the key strengths of our study is that it focuses not only on norovirus detected in individual acute gastroenteritis cases, but also on stool samples from both suspected and confirmed foodborne outbreaks. While many existing reports concentrate on norovirus genotypes from clinical patients, our approach provides deeper insights into how contaminated food may act as a critical transmission route. This dual emphasis on person-to-person and foodborne transmission enhances our understanding of norovirus epidemiology, offering a valuable baseline for future studies aimed at developing targeted public health interventions.

In conclusion, our analysis underscores that GII.2[P16] remains a major driver of norovirus infections in childcare centers and schools in Korea, highlighting the vulnerability of densely populated and immunologically susceptible groups. The emergence and sustained circulation of GII.4 variants also warrant ongoing monitoring, given their notable role in global norovirus outbreaks. Additionally, the observed seasonal shift, with outbreaks extending into late spring, necessitates more vigilant surveillance throughout the year. By incorporating samples from foodborne outbreaks, this study expands the current knowledge base and illustrates the importance of contaminated food as a transmission vector to inform better prevention strategies. To effectively control norovirus infections, comprehensive measures—such as heightened hygiene management, improved surveillance systems, and the development of vaccines targeting prevalent genotypes—must be pursued. Further molecular epidemiological research will be crucial for unraveling the evolving trends and rout es of norovirus transmission in group settings.

## Figures and Tables

**Table 1:** Primers and probes used for NoV detection by real-time and conventional RT-PCR.

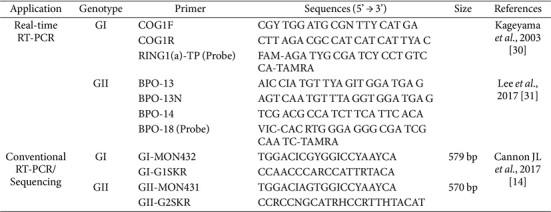

**Table 2:** Summary of norovirus genotypes.

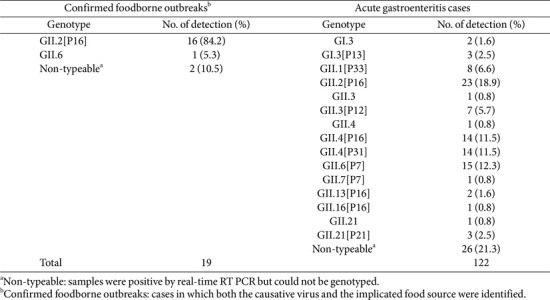

**Table 3:** Distribution of Norovirus Genotypes by Age and School Type.